# Closed-Loop Chemical Recycling of Poly(butylene succinate) Using Organocatalysts

**DOI:** 10.3390/polym18111267

**Published:** 2026-05-22

**Authors:** Na Liu, Peng Du, Yi Meng, Gangqiang Zhang, Kaitao Zhang, Yu Pan

**Affiliations:** 1 Institute of Functional Textiles and Advanced Materials, College of Textiles and Clothing, Qingdao University, Qingdao 266071, China; anlanan0212@163.com (N.L.);; 2 College of Textiles and Clothing, Qingdao University, Qingdao 266071, China

**Keywords:** depolymerization, poly(butylene succinate), organocatalyst, closed-loop recycling

## Abstract

Plastics are indispensable to modern life, yet pose a double-edged sword as their escalating production threatens human health and ecosystems. This urgent reality drives intensive efforts to develop recycling technologies that convert waste plastics into valuable feedstocks. Herein, we develop an efficient organocatalytic strategy for the depolymerization and closed-loop chemical recycling of poly(butylene succinate) (PBS). The strong organic base TBD demonstrated the highest catalytic activity for the methanolysis depolymerization of PBS, achieving a yield of 93.1% under mild conditions (100 °C, 2 h). GC and MS analyses identified dimethyl succinate (DMS) and 1,4-butanediol (1,4-BDO) as the major products. Investigation into the depolymerization behavior and mechanism revealed that the process proceeds via random chain scission, facilitated by a dual hydrogen-bonding activation mechanism mediated by TBD. Closed-loop chemical recycling was achieved by repolymerizing the recovered monomers into PBS. The reproduced polymer exhibited properties comparable to commercial virgin PBS. Moreover, this strategy could be extended to other commercial polyester systems, establishing an eco-friendly and viable pathway for sustainable polymer recycling.

## 1. Introduction

Plastics have become an indispensable material in modern society, with global annual consumption reaching 460 million tons and projected to triple by 2050 [1], presenting significant challenges regarding oil dependence and carbon emissions [2,3,4,5,6,7]. To alleviate the environmental and resource issues, aliphatic polyesters such as poly(lactic acid) (PLA), poly(ε-caprolactone) (PCL), and poly(butylene succinate) (PBS) have been widely adopted as promising alternatives due to their superior biodegradability and biocompatibility [8,9,10,11,12,13,14]. However, in conventional end-of-life treatments such as composting or energy recovery, carbon resources are merely converted or dissipated rather than retained, which does not enable closed-loop material cycling [15,16,17,18,19]. Consequently, chemical recycling has emerged as a promising approach for converting waste plastics into valuable monomers, providing a viable pathway toward sustainable resource management and a closed-loop economy [20].

As a transformative strategy for sustainability, chemical recycling to monomer (CRM) is recognized not only as a key method for plastic waste management but also as an increasingly essential component of the circular economy [21]. CRM employs depolymerization processes to break down waste polymers into their constituent monomers. As a prerequisite for closed-loop chemical recycling, CRM enables the regeneration of high-purity monomers that can be re-polymerized into virgin-grade plastics [22]. In recent years, CRM has attracted considerable attention due to its selective decomposition of polymer waste, environmental benefits, and its potential to produce plastics with qualities comparable to virgin materials [23]. Among various polymer systems, poly(ethylene terephthalate) (PET) has served as a representative model for CRM due to its industrial relevance and well-defined depolymerization chemistry [24]. Significant advances in PET methanolysis [25,26] and glycolysis [27,28] have been achieved through catalytic system optimization. These studies collectively underscore the importance of catalyst design and thermodynamic regulation in enhancing efficiency and selectivity. Inspired by hydrolases, a biomimetic dinuclear Zn complex was reported for PET depolymerization, exhibiting catalytic activities ranging from milligram to hundred-gram scales under alkaline conditions (pH 8–13) and at 40–90 °C [29]. The mechanism was presented that the biomimetic Zn–Zn sites activated the plastic, stabilized the key intermediate and enabled intramolecular hydrolysis. The bio-based catalytic system based on guaiacol and potassium bicarbonate was reported to depolymerize PET under mild conditions (120 °C, 0.6 MPa), achieving 94% dimethyl terephthalate (DMT) and 98% ethylene glycol (EG) yields within 2 h [30]. This system also showed versatility for various polyesters and real-world PET waste streams, including mixed textiles and colored plastics. Similar catalytic strategies have been applied to other commercial polyesters. Thermally robust Sn(II)-based complexes have demonstrated efficient depolymerization of PLA under industrially relevant conditions, highlighting the necessity of catalyst stability in high-temperature CRM processes [31]. In such systems, catalytic approaches are considered indispensable for enabling controlled depolymerization under practical conditions, whereas simple thermal degradation often suffers from poor selectivity and limited controllability [32,33,34].

In comparison to the commodity thermoplastic PET, PBS is a biodegradable and biocompatible polyester developed in recent decades for applications in packaging, agricultural films, disposable tableware, and biomedicine [35,36,37,38,39]. In 2025, the global production capacity of PBS reached 33,000 tons/year [40]. Despite effective PBS degradation via compost [41,42] and enzymatic methods [43,44], studies focusing on catalytic routes to monomer recovery represents an effective strategy for achieving closed-loop recycling and facilitating resource recovery [45,46]. The selective depolymerization of several commercial plastics under mild conditions was enabled by employing zinc bis[bis(trimethylsilyl)amide] (Zn(HMDS)_2_) as a catalyst [47]. Meanwhile, lanthanum acetylacetonate (La(acac)_3_) was reported to use as the catalyst in the methanolysis of PBS, yielding 99% dimethyl succinate after 4 h at 90 °C [48]. An alkaline hydrolysis method enabling closed-loop recycling of PBS was established, achieving a depolymerization yield of 97.3% under the conditions of 140 °C, 4 h, and 4 mmol of NaOH [49]. Recently, the depolymerization of PBS catalyzed by a zinc complex based on a multidentate ligand was reported to achieve up to 98% conversion with a 62% yield of dimethyl succinate after 48 h in solution, whereas solvent-free methanolysis reached high conversion within 1 h at elevated temperature [50]. However, although the depolymerization of PBS has been demonstrated for zinc and other metal-based systems, highly efficient depolymerization process utilizing organic catalyst, particularly for the closed-loop recycling of PBS, is still very limited [51,52]. Especially, a recent study reported that a tertiary amine-catalyzed methanolysis process was able to depolymerize both fossil fuel- and biobased polyesters, including PET, PLA, poly(butylene adipate-co-terephthalate) (PBAT) and PBS in one reactor under mild conditions with high monomer yields [52]. Herein, we report an organocatalytic system for the depolymerization of PBS, enabling its chemical recycling to monomer and closed-loop recovery. Following depolymerization, the monomers dimethyl succinate (DMS) and 1,4-butanediol (1,4-BDO) could be collected, and subsequently repolymerized into PBS with the same structure through transesterification-polycondensation process. The study of depolymerization behavior was found to be a random chain-scission process. Furthermore, this organocatalytic system could also depolymerize post-consumer PBS products, PET, PLA and PCL. This work provides a metal-free, value-added recycling path to achieve sustainable material circularity for biodegradable polyesters.

## 2. Materials and Methods

### 2.1. General Procedures and Materials

All air- and moisture-sensitive reactions were conducted under dry argon atmosphere using standard Schlenk and/or glovebox techniques. Commercially available PBS was sourced as industrial-grade pellets and was directly employed in the depolymerization experiments without any additional pretreatment. Catalysts were purchased from commercial sources and used directly without further purification. Methanol (MeOH, water content ≤ 30 ppm) used for depolymerization was obtained from J&K Scientific Ltd. (Beijing, China) All other chemicals were commercially available and used as received without further purification. In addition, PET, PLA, and PCL products were obtained from commercial sources, washed and dried, and then transferred into the glovebox for direct use.

General procedure for catalyzed PBS depolymerization: In a glovebox, PBS (860 mg), catalyst (1 mol%), and methanol (10 equiv.) were added to an argon-filled Schlenk flask. The mixture was then stirred at 100 °C for 1 h to achieve efficient depolymerization. After completion of the reaction, the reaction mixture was analyzed by ^1^H NMR spectroscopy to determine the yield of DMS.

General procedure for PBS polycondensation: In the first step, transesterification was performed at 220 °C under a nitrogen atmosphere using 1 mol% Ti(OBu)_4_ as the catalyst with stirring (~300 rpm). Methanol generated during the reaction was continuously removed by distillation, and the reaction was considered complete when no further distillate was observed. Subsequently, the temperature was increased to 240 °C, and melt polycondensation was conducted under reduced pressure (2–5 mbar) for 2–3 h to obtain regenerated PBS.

### 2.2. Characterization and Measurements

The nuclear magnetic resonance (NMR) spectra were collected by a Bruker AVANCE III HD 400 spectrometer (Bruker, Karlsruhe, Germany).

Gas chromatography (GC) analysis was conducted using a GC9790-II instrument manufactured by Fuli Corporation (Taizhou, China), equipped with an RB-5 quartz capillary column (30 m in length, 0.32 mm inner diameter, 0.25 µm film thickness). High-purity nitrogen was employed as the carrier gas.

High-resolution mass spectrometry (HRMS) was conducted on a Q Exactive Orbitrap mass spectrometer (Thermo Fisher Scientific). Samples were dissolved in methanol and analyzed by electrospray ionization (ESI) in positive or negative ion mode to obtain accurate molecular ion peaks (*m*/*z*) and isotopic distributions.

Gel permeation chromatography (GPC) was performed using a Waters 2414 refractive index detector (Waters, Milford, MA, USA) to determine the relative molecular weight and its distribution of the polymers. CDCl_3_ was used as the eluent at a flow rate of 1.0 mL/min, and both the column and detector temperatures were maintained at 40 °C. The samples were dissolved in the eluent, filtered, and then injected for analysis to obtain the number-average molecular weight (*M*_n_), weight-average molecular weight (*M*_w_), and polydispersity index (PDI).

Scanning electron microscopy (SEM) images were obtained on JSM-6390LV instrument (JEOL). Samples were mounted on specimen stubs and sputter-coated with gold prior to analysis. Images were recorded at an accelerating voltage of 15 kV with magnifications of 500× and 1000×.

Infrared (IR) spectra were recorded by a Nicolet iS50 FT-IR spectrometer (Thermo Fisher Scientific, Waltham, MA, USA) using the attenuated total reflection mode in the wavelength range of 500–4000 cm^−1^.

X-ray diffraction (XRD) patterns were recorded using a SmartLab 3 kW diffractometer (JEOL, Tokyo, Japan) with Cu Kα radiation (λ = 0.154 nm) operated at 40 kV and 200 mA. The data were collected over a 2θ range of 5–60° at a scanning rate of 10° min^−1^.

Differential scanning calorimetry (DSC) was performed using a DSC 2500 differential scanning calorimeter (TA Instruments, New Castle, DE, USA) under a nitrogen atmosphere. The melting and crystallization behaviors of DMS were analyzed over a temperature range of −50 to 150 °C at a heating rate of 10 °C min^−1^.

Thermogravimetric analysis (TGA) was carried out using a TGA5500 thermogravimetric analyzer (TA Instruments, New Castle, DE, USA). The samples were heated from room temperature to 700 °C at a rate of 10 °C min^−1^ under a nitrogen atmosphere to obtain the thermogravimetric curves.

## 3. Results and Discussion

### 3.1. Catalyst Screening and Catalytic Performance

As one of the most important depolymerization methods, methanolysis of PBS generates DMS and 1,4-BDO, which can be readily repolymerized into new polymers via condensation polymerization [53]. The industrial-grade PBS pellets (*M*_n_ = 57.1 kg/mol, PDI = 1.79) were used as the model polymer to investigate the depolymerization by a series of Lewis basic catalysts (Table 1). The degree of depolymerization was estimated based on the yield of DMS, which was determined by ^1^H NMR spectroscopy using signal integrations normalized according to the number of protons of the corresponding resonances, specifically the methoxy protons of DMS (δ ≈ 3.70 ppm) and the methylene protons of the PBS backbone (δ ≈ 4.10 ppm). At first, three common strong organic bases, 4-dimethylaminopyridine (DMAP), 1,5,7-triazabicyclo[4.4.0]dec-5-ene (TBD) and 1,8-diazabicyclo[5.4.0]undec-7-ene (DBU), were employed as catalysts for the depolymerization reaction, owing to their highly basic nature and excellent catalytic activity in organic reactions [54,55,56,57,58] and polymerization [59,60,61,62,63]. With 10 equivalents of methanol, the DMAP-based system (1 mol%) afforded only 4.4% yield at 100 °C (Table 1, run 1). Meanwhile, a yield of 85.8% was observed with 1% TBD under the same conditions, exhibiting much better catalytic performance (Table 1, run 2). In comparison, the strong organic base DBU showed a yield of 55.6% (Table 1, run 3). Meanwhile, the other three strong organic bases, 1,5-diazabicyclo[4.3.0]non-5-ene (DBN), tert-butylimino-tri(pyrrolidino)phosphorene (P_1_-*^t^*Bu) and N,N-dimethylimidodicarbonimidic diamide (metformin), only exhibited moderate activity, with yields of 40.5%, 47.2% and 54.0%, respectively (Table 1, runs 4–6). In addition, the derivative of TBD, 7-methyl-1,5,7-triazabicyclo[4.4.0]dec-5-ene (MTBD), showed a yield of 77.3%, which was a bit lower than that of TBD. This might be attributed to the substituted methyl in MTBD, which weakens its hydrogen-bond donor ability and thereby reduces cooperative activation of the ester bond compared with TBD owing to the steric hindrance. In comparison to these organic bases, the depolymerization of PBS was also attempted using the strong metal alkoxide base potassium tert-butoxide (*^t^*BuOK). This reaction exhibited 86.2% yield at 100 °C, similar to the result obtained with TBD. This comparable yield suggests that TBD possesses strong catalytic activity toward ester bond cleavage, achieving performance similar to that of a strong alkoxide base. However, the Lewis acids, Zn(OAc)_2_ and ZnCl_2_, only exhibited relatively lower catalytic activities (Table 1 runs 9 and 10) for the depolymerization of PBS under the same yields, indicating that basic catalysts were probably more effective for PBS depolymerization. Based on the preliminary exploration, TBD was employed as a representative catalyst for the depolymerization of PBS under various conditions. When the reaction time was extended to 2 h, the depolymerization catalyzed by TBD showed a slightly higher yield of 93.1% (Table 1, run 11). The small amount of incomplete depolymerization product may be due to residual linear or cyclic oligomers [51,64]. Compared with reported catalytic systems, the Zn(HMDS)_2_ system requires 9 h at 100 °C to achieve an 83% DMS yield [47], while the NaOH-based hydrolysis method can reach approximately a 90% DMS yield under relatively harsh conditions (140 °C, >4 h) [49]. In contrast, the present system enables efficient depolymerization of PBS under milder conditions and within a shorter reaction time, while achieving a comparable DMS yield to those reported systems, thus demonstrating excellent catalytic performance. As the loading of TBD decreased from 1% to 0.2%, a significant reduction in catalytic activity was observed (Table 1, runs 12 and 13). Likewise, when the reaction temperature was lowered to 60 °C, a significant decrease in its depolymerization activity was also observed (Table 1, run 14). Increasing the amount of methanol was able to slightly increase the conversion (Table 1, run 15).

### 3.2. Analysis of Depolymerization Products

**Figure 1:**
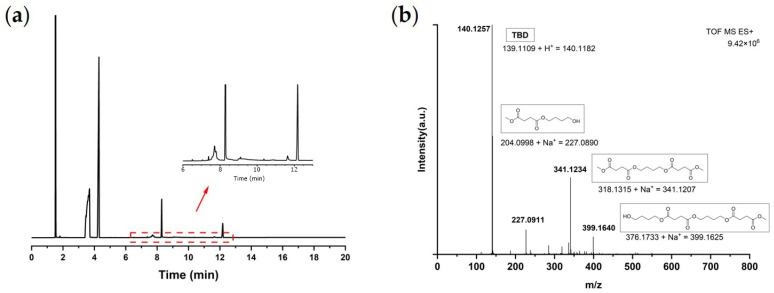
(**a**) GC and (**b**) HRMS analysis of products from the TBD-catalyzed degradation of PBS.

The GC was employed to analyze the resultant compounds from the depolymerization process. It was shown that the major products of the depolymerization were identified as DMS and 1,4-BDO (Figure 1a), as evidenced by the matching of their GC retention times with those of pure reference samples (Figure S17). The retention times of DMS and 1,4-BDO were approximately 3.624 min and 4.292 min, respectively, matching well with those of the corresponding peaks in the sample. This observation is consistent with the anticipated outcome of the depolymerization via methanolysis [65]. The additional chromatographic peaks can be attributed to the formation of dimer or trimer compounds. In order to analyze the products of depolymerization in detail, we performed HRMS analysis on the post-depolymerization mixture. The presence of dimers and trimers was detected in the mass spectrum (Figure 1b), that also corroborated the results obtained from the GC chromatogram. However, the depolymerization products, DMS and 1,4-BDO, failed to be detected in the mass spectrum, possibly due to their low molecular weights which made them not amenable to the current analytical method. The presence of residual oligomers is consistent with the observation by ^1^H NMR that depolymerization could not be proceed to completion.

### 3.3. Depolymerization Behavior and Structural Evolution of PBS

**Figure 2:**
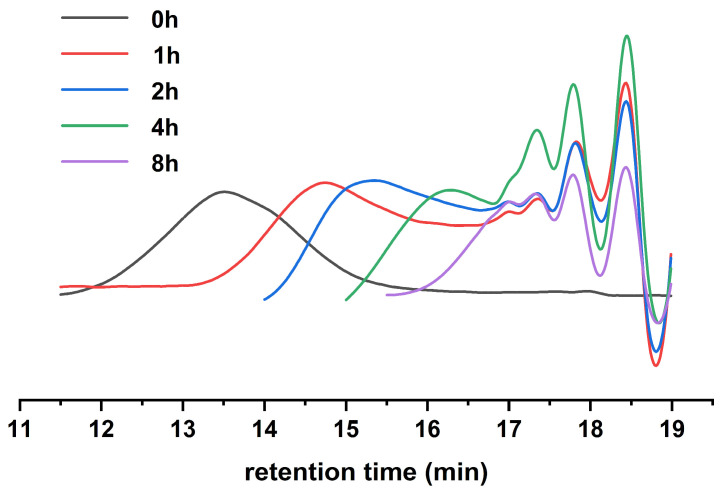
GPC curves recorded at different time points for TBD-catalyzed degradation of PBS at 80 °C.

**Figure 3:**
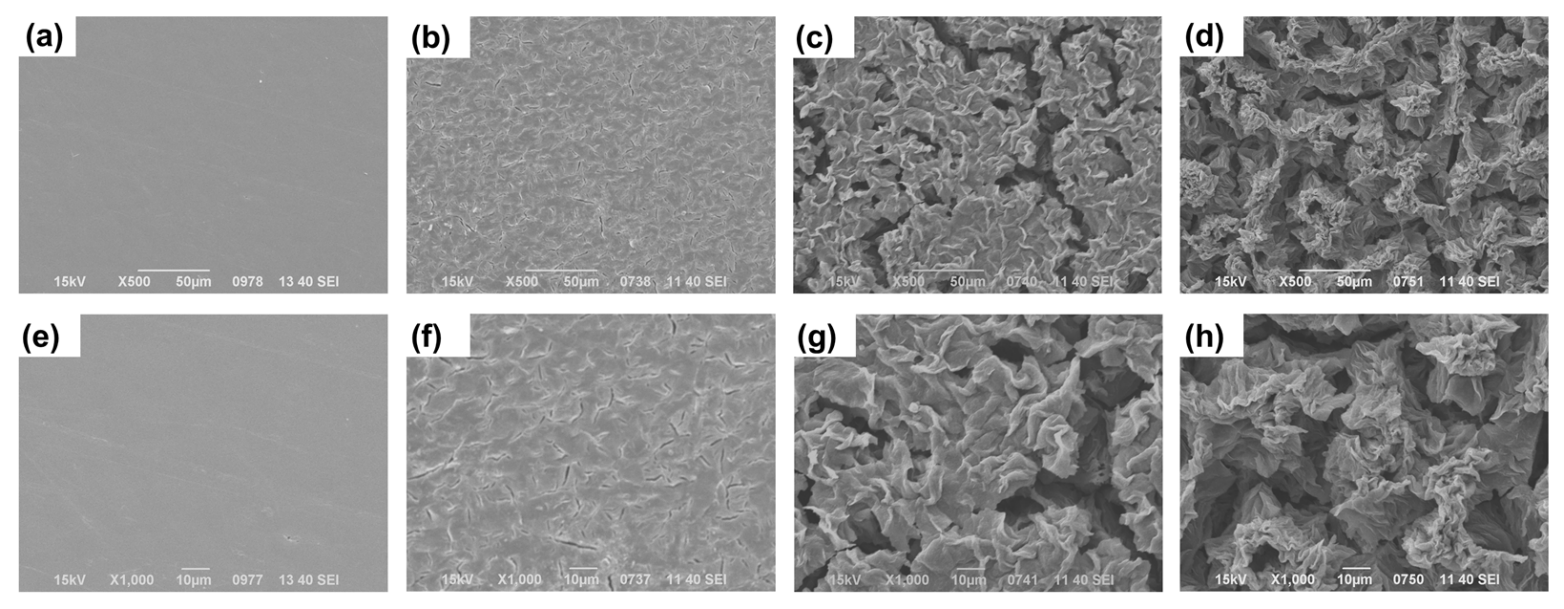
SEM micrographs showing the surface morphology of PBS sheets at magnifications of 500× and 1000×, and at different time points during TBD-catalyzed degradation at 100 °C: pristine (**a**) 500× and (**e**) 1000×; after 2 min of reaction (**b**) 500× and (**f**) 1000×; after 5 min of reaction (**c**) 500× and (**g**) 1000×; after 15 min of reaction (**d**) 500× and (**h**) 1000×.

In order to detect the depolymerization behavior, the relationship between the degree of depolymerization and molecular weights was investigated according to the ^1^H NMR and GPC analysis. The reaction progress was monitored in real-time by withdrawing aliquots at specific intervals. The degree of depolymerization could be evaluated by calculating the ratio of the integral area of the characteristic methyl proton peak adjacent to the ester group in the DMS depolymerization product to the integral area of the characteristic methylene proton peak of the PBS chain repeating unit. With the progress of the depolymerization reaction, the degree of depolymerization progressively increased according to the ^1^H NMR spectra (Figure S18, Table S1). Meanwhile, the GPC elution profiles of the residual polymers gradually shifted toward lower molecular weights and broadened with multiple peaks at longer reaction times (Figure 2). This might be attributed to the presence of a mixture of discrete oligomers. The appearance of shoulder peaks or minor distributions further indicates the formation of oligomers. Although these oligomers remain minor relative to the main distribution, they still contribute to the overall dispersity. In addition, the negative signal observed at approximately 18.5–19 min was attributed to solvent mismatch between residual methanol in the sample and chloroform as the mobile phase. Since methanol has a lower refractive index than chloroform, this results in a negative response in the refractive index detector (Figure S19). These results indicated that the depolymerization of PBS catalyzed by TBD was found to be a random chain-scission process. During the depolymerization process, the SEM analysis was also used to monitor the surface features of PBS sheets at various stages (Figure 3). As degradation progressed, the surface of the PBS sheets evolved from smooth to increasingly rough as a result of continuous erosion. This multi scale structural evolution enhanced the specific surface area of the PBS sheets, thereby providing more favorable conditions for subsequent depolymerization reactions. The observed morphological progression appeared to be consistent with a degradation behavior involving random chain scission.

### 3.4. Proposed Depolymerization Mechanism of PBS

**Figure 4:**
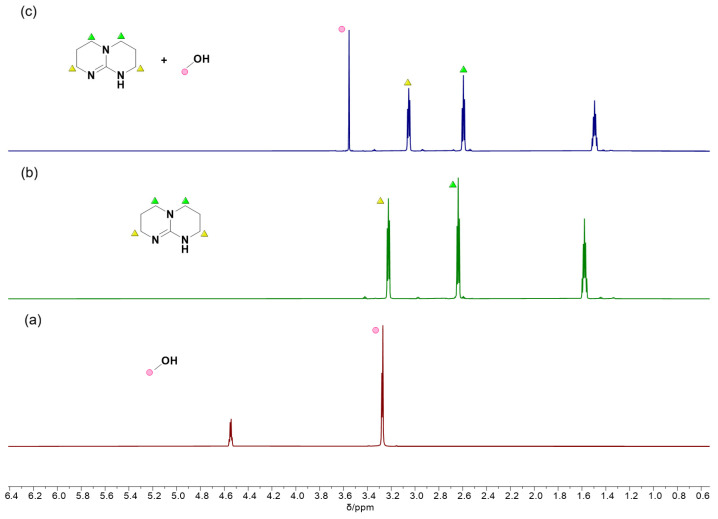
Stacked ^1^H NMR spectra (C_6_D_6_, 500 MHz, 25 °C) of (**a**) methanol, (**b**) TBD, and (**c**) the mixture with equivalent amounts of TBD and methanol.

**Scheme 1:**
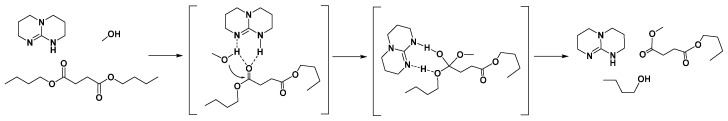
The possible pathway for depolymerization of PBS by TBD.

**Figure 5:**
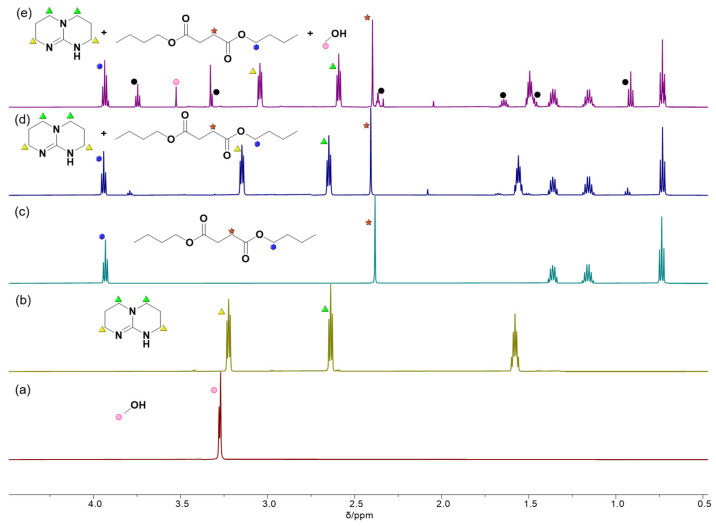
Stacked ^1^H NMR spectra (C_6_D_6_, 500 MHz, 25 °C) of (**a**) methanol, (**b**) TBD, (**c**) dibutyl succinate, (**d**) the mixture with equivalent amounts of TBD and dibutyl succinate, and (**e**) the mixture with equivalent amounts of TBD, dibutyl succinate and methanol.

The depolymerization mechanism was further investigated by in situ monitoring of simulated small molecule reactions using NMR spectroscopy. Initially, TBD was mixed with an equivalent amount of methanol in an NMR tube for analysis. The ^1^H NMR spectra revealed a downfield shift in the methyl resonance of methanol, from 3.26 ppm to 3.55 ppm (Figure 4). In addition, the signals corresponding to the four methylene groups adjacent to the nitrogen atom in TBD exhibited upfield shifts from 3.22 and 2.64 ppm to 3.06 and 2.59 ppm respectively. It is demonstrated that a clear hydrogen-bonding interaction forms between TBD and methanol upon mixing. Furthermore, upon mixing TBD with an equivalent amount of dibutyl succinate, a model compound for PBS, the ^1^H NMR spectra revealed slight shifts for both TBD and dibutyl succinate, indicating a weak interaction between them. ^1^H NMR analysis of the equimolar TBD/methanol/dibutyl succinate mixture showed an upfield shift in the TBD characteristic peak to 3.04 ppm. Meanwhile, the methyl resonance peak of methanol shifted from 3.26 ppm to 3.52 ppm and 3.33 ppm, suggesting the presence of different interactions with other molecules. Remarkably, a set of characteristic peaks observed in the ^1^H NMR spectrum could be assigned to new dibutyl succinate molecules (black dots in Figure 5e), based on their integration ratio and splitting pattern, which might be attributed the interaction with methanol and TBD [66,67]. The integral ratios of the characteristic peaks for methanol and dibutyl succinate are each approximately 1:3. In contrast, the ^1^H NMR spectrum of the equimolar DMAP/methanol/dibutyl succinate mixture showed no new signals or distinct shifts for dibutyl succinate (Figure S20), while only a distinct shift in the methyl signal of methanol was observed. This indicates that DMAP activates methanol via hydrogen bonding, rather than dibutyl succinate, which probably explains its inferior catalytic performance on depolymerization compared to TBD. Based on the experimental observations, a depolymerization pathway for PBS by TBD could be proposed, which followed a dual activation mechanism mediated by hydrogen bonding, as illustrated in Scheme 1 [67].

### 3.5. Closed-Loop Chemical Recycling of PBS

**Figure 6:**
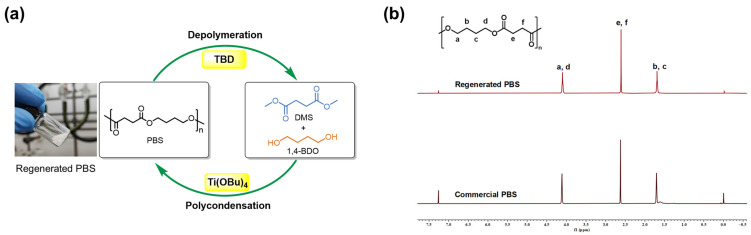
(**a**) Closed-loop recycling process of PBS; (**b**) ^1^H NMR spectra of commercial and regenerated PBS.

**Figure 7:**
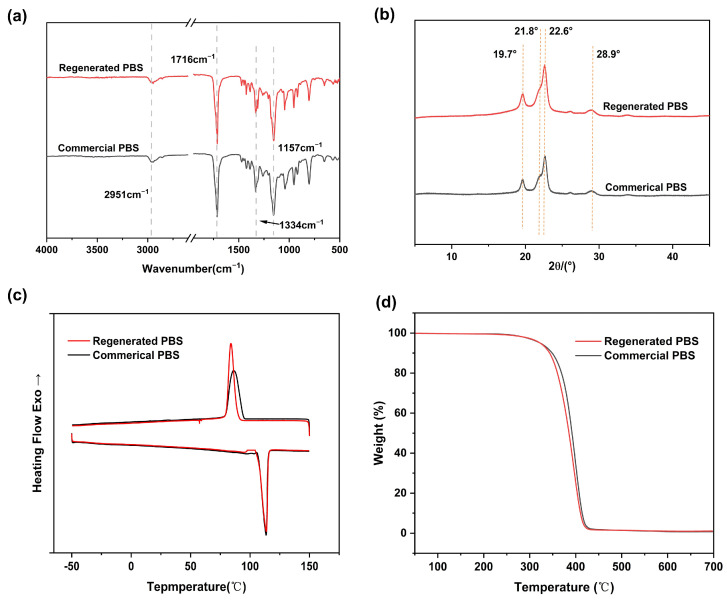
(**a**) FTIR spectra of commercial and regenerated PBS; (**b**) XRD patterns of commercial and regenerated PBS; (**c**) DSC curves of commercial and regenerated PBS; (**d**) TGA curves of commercial and regenerated PBS.

Closed-loop recycling allows plastic waste to be converted back to its original state, providing a critical strategy for preserving polymer performance and achieving resource circularity [68]. Based on a study of PBS depolymerization using an organic base, we attempted to explore the separation of the resulting products and their subsequent repolymerization to establish a closed-loop recycling route. Given that 1,4-BDO (b.p. 231 °C at 1 atm) [69] and DMS (b.p. 200 °C at 1 atm) [70] have similar boiling points, the depolymerized mixture was subjected to vacuum distillation. This process afforded a liquid mixture of 1,4-BDO and DMS in a total yield of 86%, with a composition ratio of approximately 1:1 as determined by GC analysis. The polymerization of recycled DMS and 1,4-BDO was catalyzed by 1 mol% tetrabutyl titanate (Ti(OBu)_4_) via a transesterification-polycondensation process producing the regenerated PBS as a white solid (Figure 6a) [71]. The regenerated PBS exhibited a *M*_n_ of 50.5 kg/mol as determined by GPC. It showed the same characteristic ^1^H NMR peaks (4.05 and 1.82 ppm) and FT-IR absorptions (2951, 1716, 1334, and 1157 cm^−1^), as well as similar XRD patterns with characteristic diffraction peaks (2θ ≈ 19.7°, 21.8°, 22.6°, and 28.9°) (Figure 6b and Figure 7a,b), confirming the same chemical structures. The DSC analysis showed identical melting and crystallization temperatures for the regenerated and commercial PBS (Figure 7c). This thermal equivalence was further confirmed by TGA, which demonstrated that their degradation behaviors were consistent (Figure 7d). These analyses demonstrate the successful attainment of closed-loop chemical recycling for PBS, from a polymer to monomer and back to a polymer.

### 3.6. Substrate Scope and Applicability of the Catalytic System

**Figure 8:**
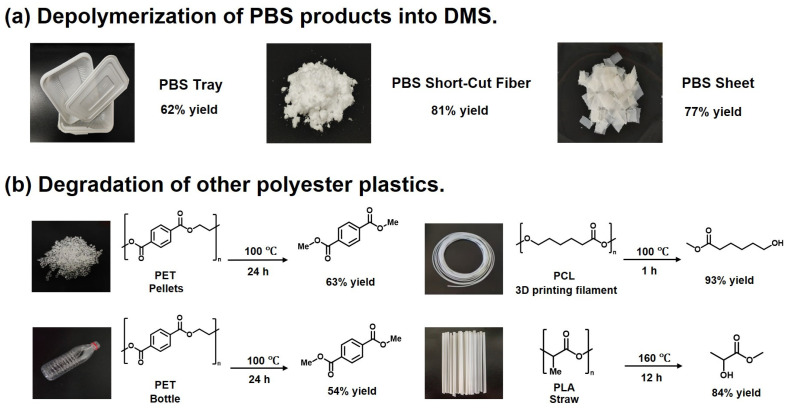
(**a**) Depolymerization of PBS products into DMS; (**b**) degradation of other polyester plastics.

Finally, the applicability of the organic base system was evaluated across some post-consumer PBS products, including trays, short-cut fibers and sheets. All materials were efficiently depolymerized with 71–86% conversion under the mild condition (100 °C, 1 h), demonstrating the adaptability and practical potential of our system (Figure 8). To further assess its versatility, the approach was extended to other polyester wastes, including PET, PLA and PCL (Figure 8b). Direct degradation of the PET pellets generated DMT in 63% yield, using the TBD catalyst under mild conditions (100 °C, 24 h). Moreover, degradation of discarded PET bottles under the same catalytic conditions (TBD, 100 °C, 24 h) afforded DMT in 54% yield. The PCL filament was efficiently degraded to give the corresponding monomer in a high yield of 93% at 100 °C for 1 h. In contrast, the PLA straw required more severe conditions (160 °C, 12 h) and afforded the product in a lower yield of 84%.

## 4. Conclusions

In conclusion, we have developed an efficient catalytic approach using organocatalysts that successfully enables the methanolysis depolymerization of PBS under mild conditions, affording DMS in a high yield. It was shown that TBD demonstrated the highest catalytic activity among the organic bases, affording DMS in up to a 93.1% yield, which was comparable to the yield obtained using the traditional strong alkoxide base *^t^*BuOK. The analysis of residuals by GC and MS analysis showed that the major products of the depolymerization were DMS and 1,4-BDO, and dimers and trimers was detected in the MS spectrum. Monitoring the reaction in real time by withdrawing aliquots at specific intervals revealed that the depolymerization of PBS proceeded via a random chain-scission process. Based on in situ NMR monitoring of the simulated small-molecule reactions, the depolymerization mechanism could be postulated to proceed via a dual hydrogen-bonding activation mechanism mediated by TBD, which simultaneously activates both the ester bond and methanol. The isolated monomer was successfully repolymerized into PBS, completing a closed-loop chemical cycle, and the properties of the regenerated polymer were virtually identical to its commercial counterpart. The demonstrated versatility of this system across PBS and other commercial polyesters (e.g., PET, PLA, PCL), enabling the recovery of corresponding monomers in moderate to high yields, establishes an environmentally friendly, closed-loop recycling pathway. This work effectively bridges the gap between sustainable polymer synthesis and practical waste management.

## Figures and Tables

**Table 1:** Depolymerization of PBS ^a^.

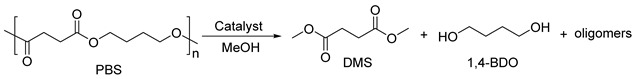				
Run	Catalyst	[PBS]:[Catalyst]	Time (h)	Yield (%) ^b^
1	DMAP	100:1	1	4.4
2	TBD	100:1	1	85.8
3	DBU	100:1	1	55.6
4	DBN	100:1	1	40.5
5	P_1_-*^t^*Bu	100:1	1	47.2
6	Metformin	100:1	1	54.0
7	MTBD	100:1	1	77.3
8	*^t^*BuOK	100:1	1	86.2
9	Zn(OAc)_2_	100:1	1	10.6
10	ZnCl_2_	100:1	1	27.5
11	TBD	100:1	2	93.1
12	TBD	500:1	16	61.3
13	TBD	150:1	9	61.0
14 ^c^	TBD	100:1	1	20.1
15 ^d^	TBD	100:1	1	87.3

^a^ Reaction conditions: PBS (860 mg, 5 mmol repeating units) n_MeOH_:n_PBSru_ = 10:1, Con._(MeOH)_ = 25 M, at 100 °C. ^b^ The yield of dimethyl succinate was determined by ^1^H NMR spectroscopy. ^c^ T = 60 °C. ^d^ n_MeOH_:n_PBSru_ = 30:1.

## Data Availability

The original contributions presented in this study are included in the article/Supplementary Materials. Further inquiries can be directed to the corresponding author.

## Supplementary Materials

The following supporting information can be downloaded at: https://www.mdpi.com/article/10.3390/polym18111267/s1, Figure S1. Quantitative 1H NMR analysis and integral normalization for DMS yield calculation; Figure S2–S16. 1H NMR spectra of the depolymerization mixtures; Figure S17–S19. The data of the study of depolymerization behavior; Table S1. The data of the study of depolymerization behavior; Figure S20. Stacked 1H NMR spectra of the mixture with equivalent amounts of DMAP, dibutyl succinate and methanol.

## Abbreviations

The following abbreviations are used in this manuscript:

PBS	poly(butylene succinate)
PLA	poly(lactic acid)
PCL	poly(ε-caprolactone)
PET	polyethylene terephthalate
PBAT	polybutylene adipate terephthalate
DMS	dimethyl succinate
1,4-BDO	1,4-butanediol
DMT	dimethyl terephthalate
EG	ethylene glycol
DMAP	4-dimethylaminopyridine
TBD	1,5,7-triazabicyclo[4.4.0]dec-5-ene
DBU	1,8-diazabicyclo[5.4.0]undec-7-ene
DBN	1,5-diazabicyclo[4.3.0]non-5-ene
P_1_-*^t^*Bu	tert-butylimino-tri(pyrrolidino)phosphorene
metformin	N,N-dimethylimidodicarbonimidic diamide
MTBD	7-methyl-1,5,7-triazabicyclo[4.4.0]dec-5-ene
*^t^*BuOK	tert-butoxide
Ti(OBu)_4_	tetrabutyl titanate
