# Supplementary material for: Closed-Loop Chemical Recycling of Poly(butylene succinate) Using Organocatalysts
Source: Polymers (Basel). 2026 May 22;18(11):1267. doi: 10.3390/polym18111267 (PMC13258939; doi:10.3390/polym18111267)
Supplement: Supplementary file 1 [file polymers-18-01267-s001.zip › polymers-4284146-supplementary.pdf]

Supporting information

Closed-Loop Chemical Recycling of Poly(butylene succinate) using Organocatalysts

Na Liu,[1] Peng Du,[2] Yi Meng,[1] Gangqiang Zhang,[1] Kaitao Zhang,[1] Yu Pan[1,*]

[1] *Institute of Functional Textiles and Advanced Materials, College of Textiles and Clothing, Qingdao University, Qingdao 266071, China.*

[2] *College of Textiles and Clothing, Qingdao University, Qingdao 266071, China.*

*Corresponding author. E-mail: ypan@qdu.edu.cn

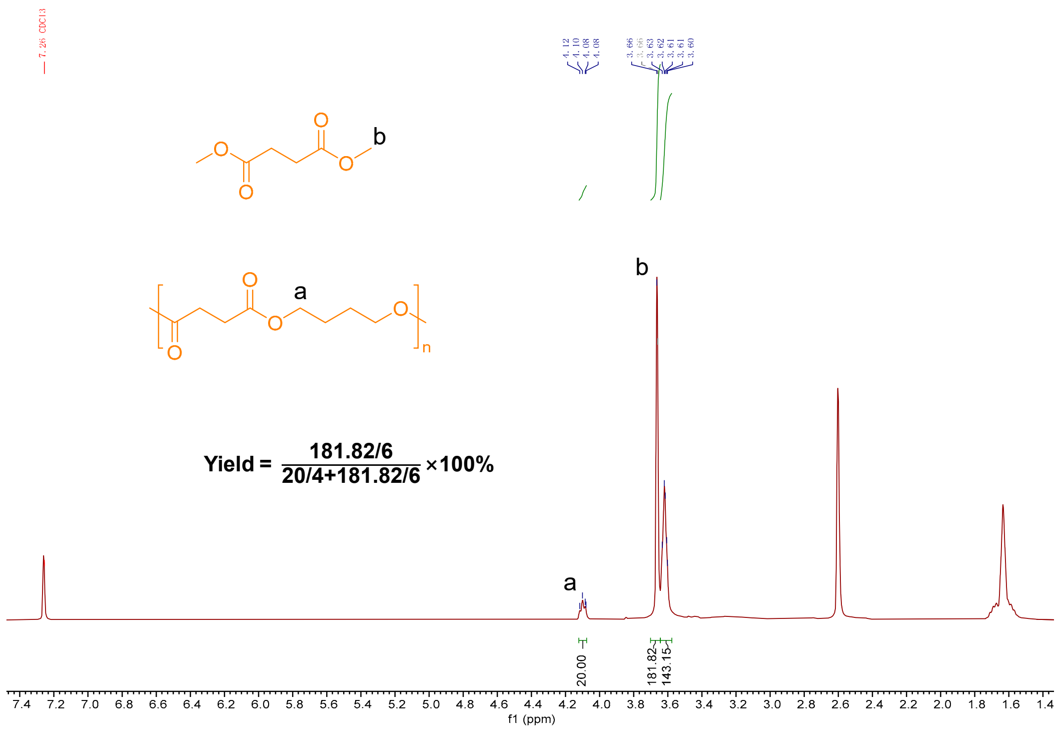

Figure S1. Quantitative ^{1}H NMR analysis and integral normalization for DMS yield calculation. (400 MHz, $CDCl_3$)

Calculation of DMS yield: The DMS yield was determined by quantitative ^{1}H NMR analysis using the methoxy protons of DMS (-OCH_3, 6H, $\delta \approx 3.70$ ppm) and the methylene protons of PBS (-OCH_2-, 4H, $\delta \approx 4.10$ ppm). The integrated peak areas were first normalized according to the number of contributing protons. Specifically, the integral of the DMS signal was divided by 6, and the integral of the PBS signal was divided by 4. The DMS yield was then calculated using the following equation:

$$DMS\ yield(\%) = \frac{I_{DMS}/6}{I_{PBS}/4 + I_{DMS}/6} \times 100$$

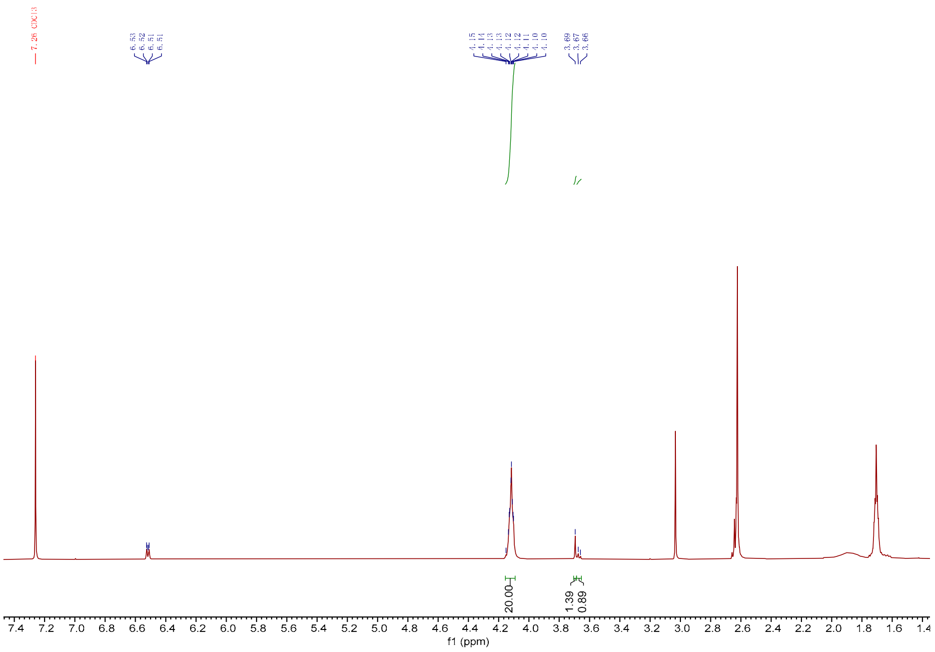

Figure S2. ^{1}H NMR (400 MHz, $CDCl_3$) spectrum of PBS after DMAP-catalyzed depolymerization.

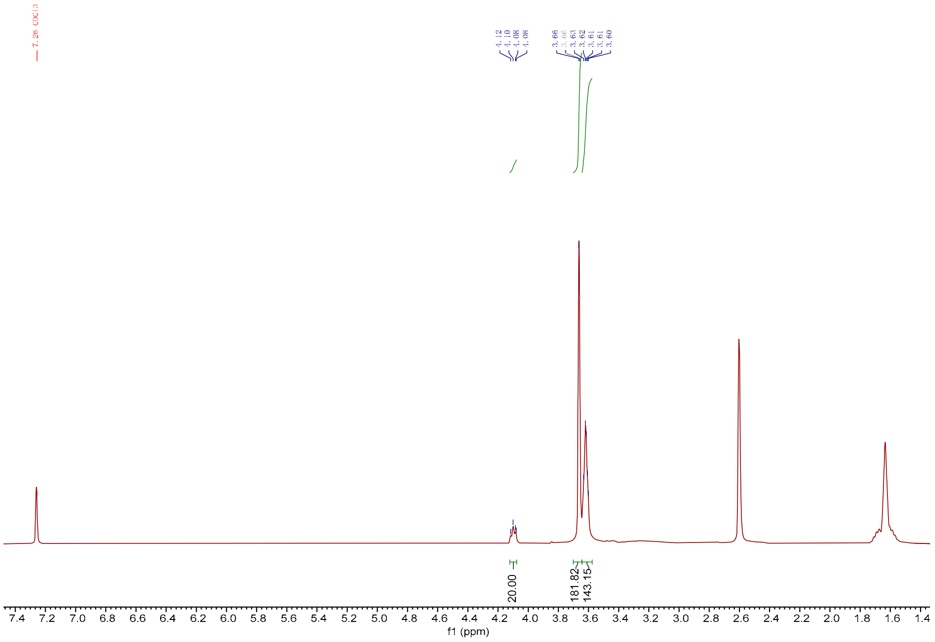

Figure S3. ^{1}H NMR (400 MHz, $CDCl_3$) spectrum of PBS after TBD-catalyzed depolymerization.

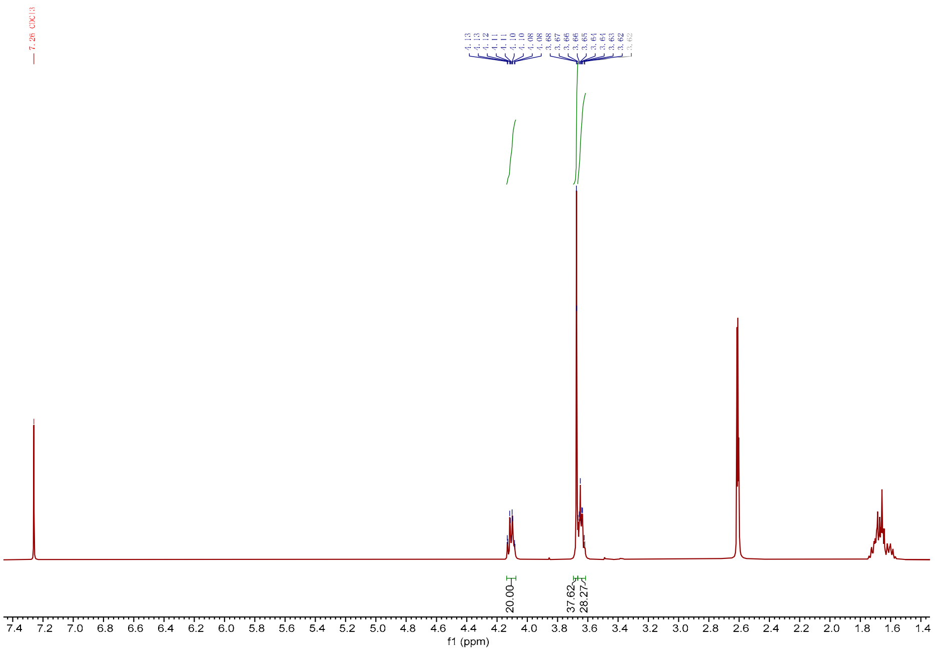

Figure S4. ^{1}H NMR (400 MHz, $CDCl_3$) spectrum of PBS after DBU-catalyzed depolymerization.

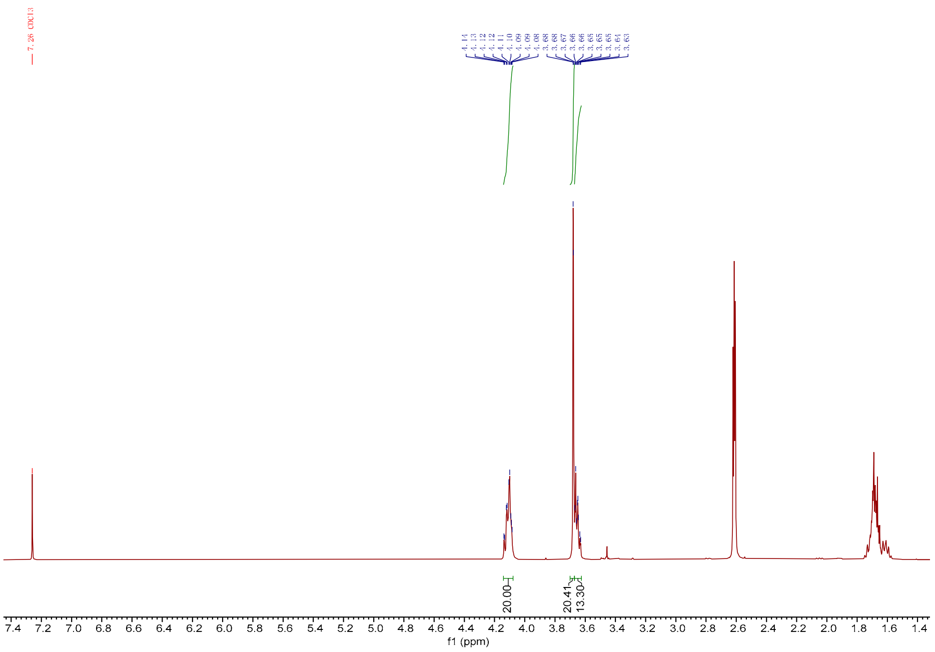

Figure S5. ^{1}H NMR (400 MHz, $CDCl_3$) spectrum of PBS after DBN-catalyzed depolymerization.

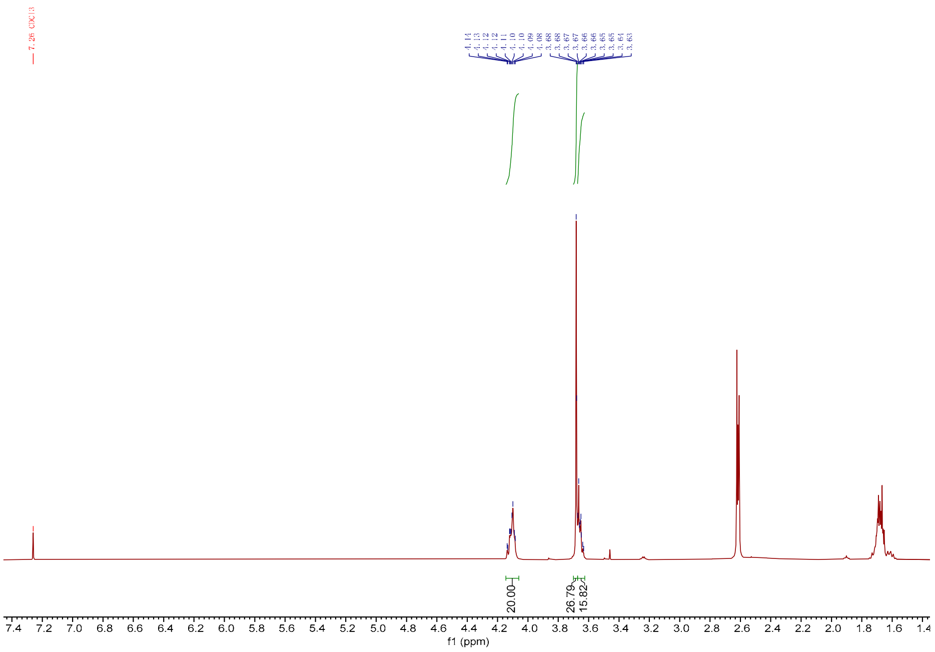

Figure S6. ^{1}H NMR (400 MHz, $CDCl_3$) spectrum of PBS after P_1-^{t}Bu-catalyzed depolymerization.

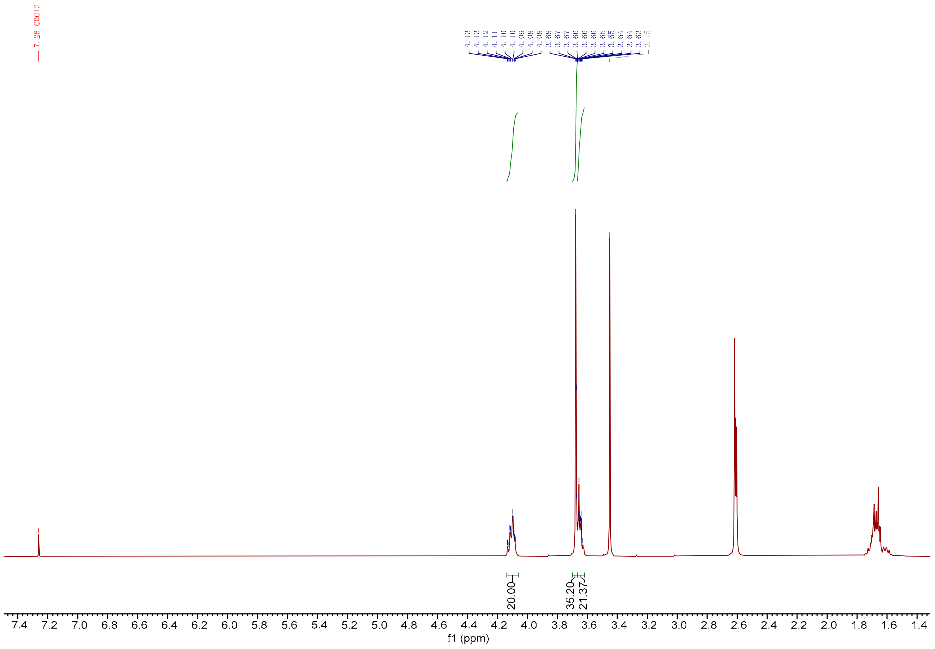

Figure S7. ^{1}H NMR (400 MHz, $CDCl_3$) spectrum of PBS after Metformin-catalyzed depolymerization.

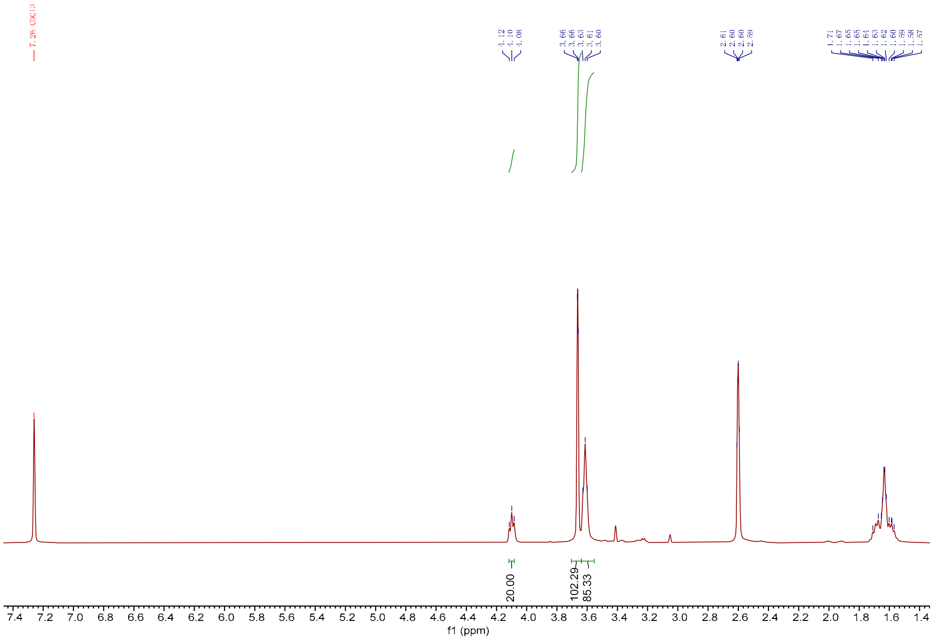

Figure S8. ^{1}H NMR (400 MHz, $CDCl_3$) spectrum of PBS after MTBD-catalyzed depolymerization.

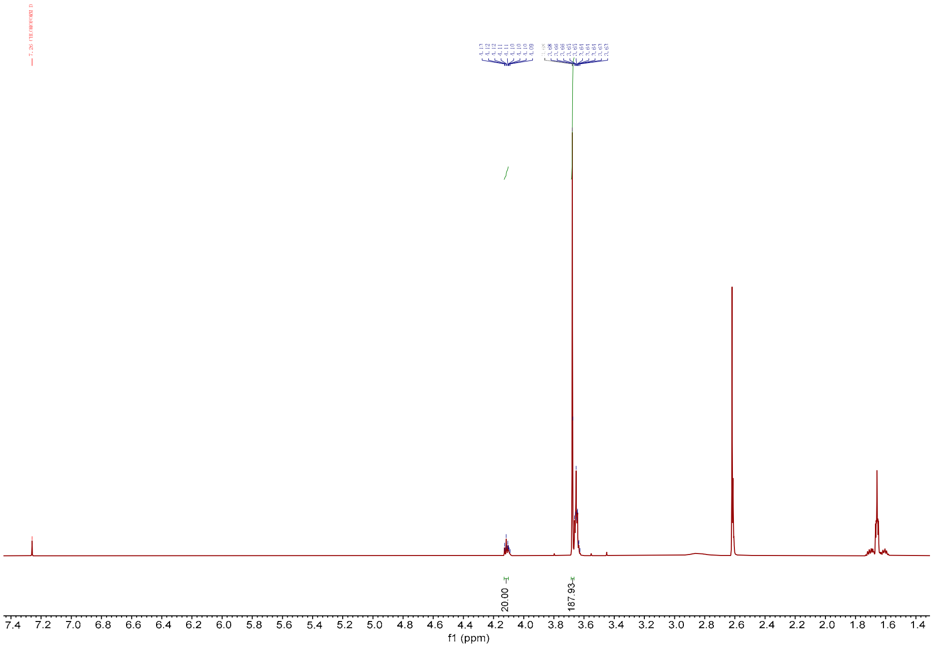

Figure S9. ^{1}H NMR (400 MHz, $CDCl_3$) spectrum of PBS after ^{t}BuOK-catalyzed depolymerization.

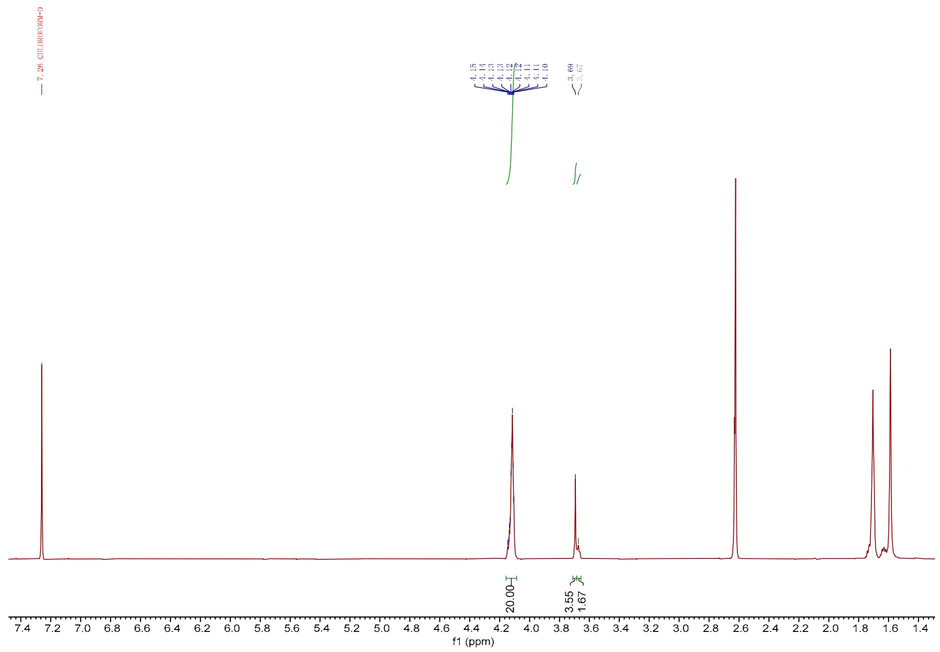

Figure S10. ^{1}H NMR (400 MHz, $CDCl_3$) spectrum of PBS after $Zn(OAc)_2$-catalyzed depolymerization.

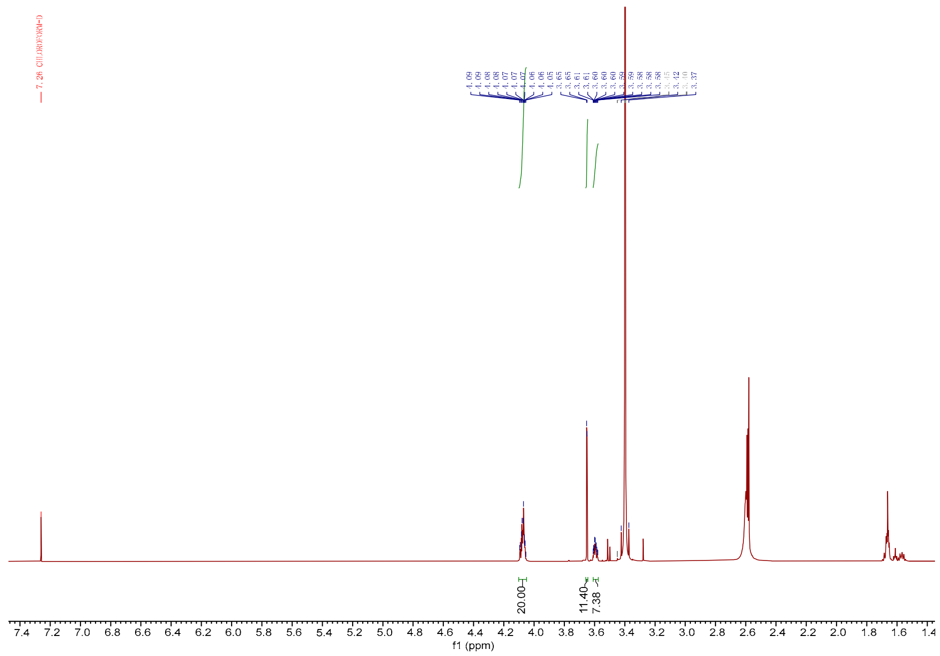

Figure S11. ^{1}H NMR (400 MHz, $CDCl_3$) spectrum of PBS after $ZnCl_2$-catalyzed depolymerization.

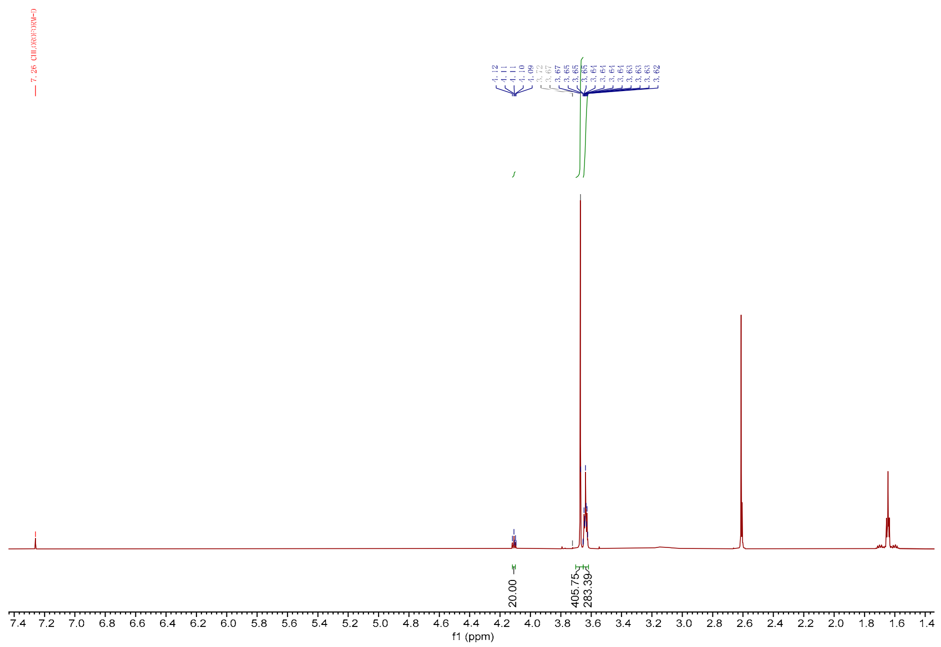

Figure S12. ^{1}H NMR (400 MHz, $CDCl_3$) spectrum of PBS after 2 h of TBD-catalyzed depolymerization.

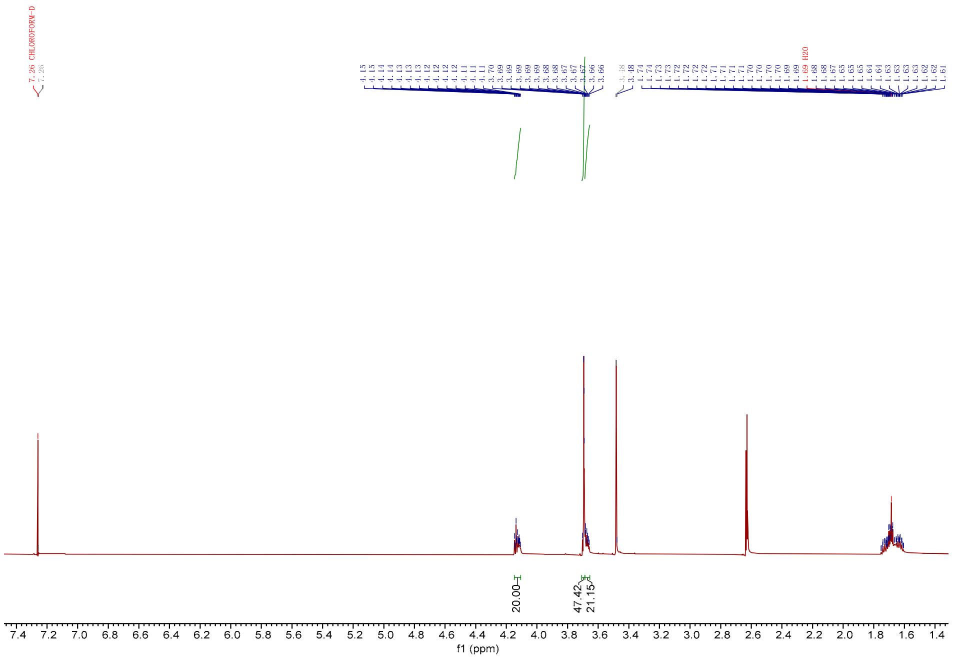

Figure S13. ^{1}H NMR (400 MHz, $CDCl_3$) spectrum of PBS depolymerization catalyzed by 0.5 mol% TBD.

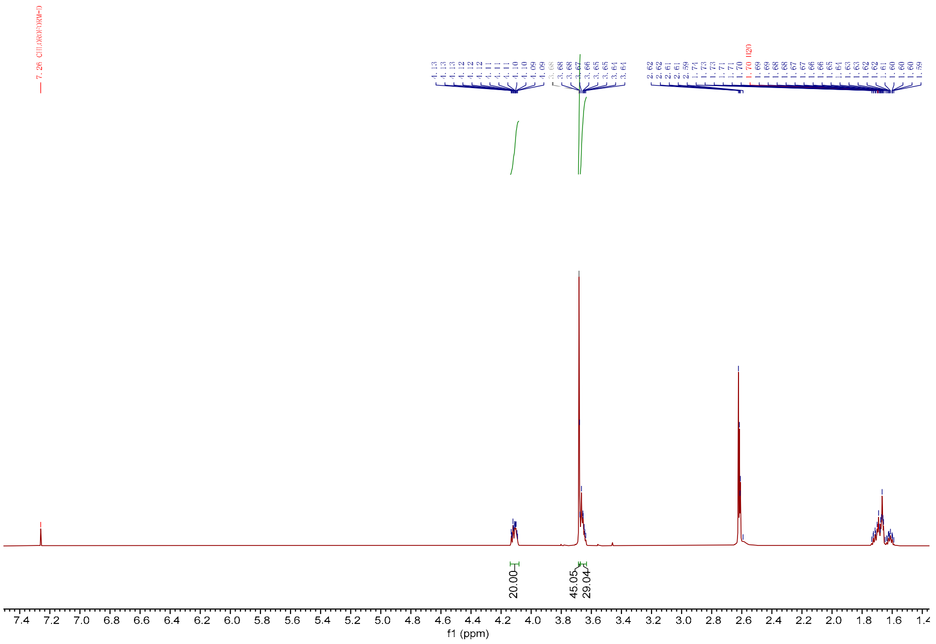

Figure S14. ^1H NMR (400 MHz, $CDCl_3$) spectrum of PBS depolymerization catalyzed by 0.8 mol% TBD.

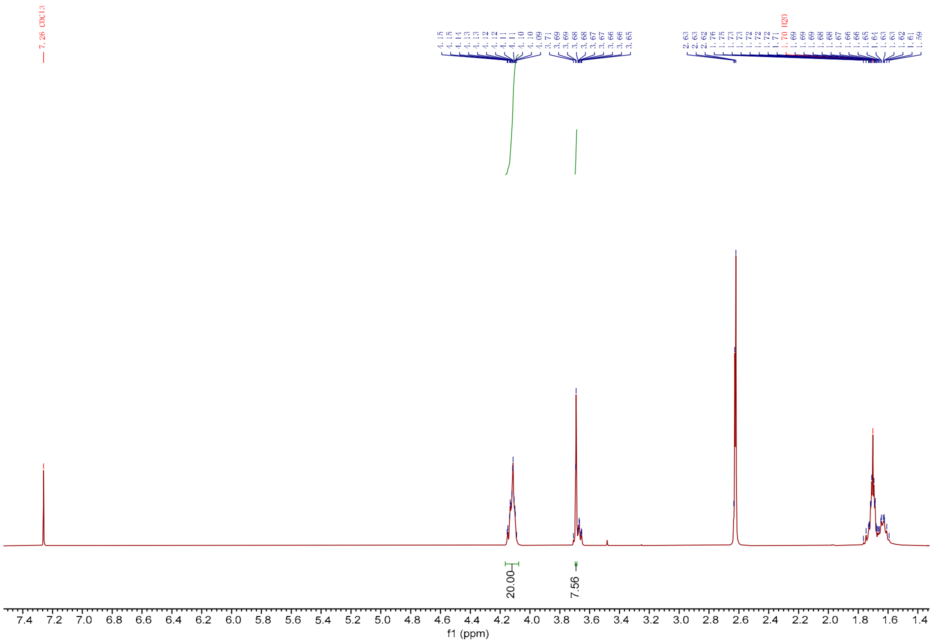

Figure S15. ^1H NMR (400 MHz, $CDCl_3$) spectrum of PBS depolymerization catalyzed by TBD at 60 °C.

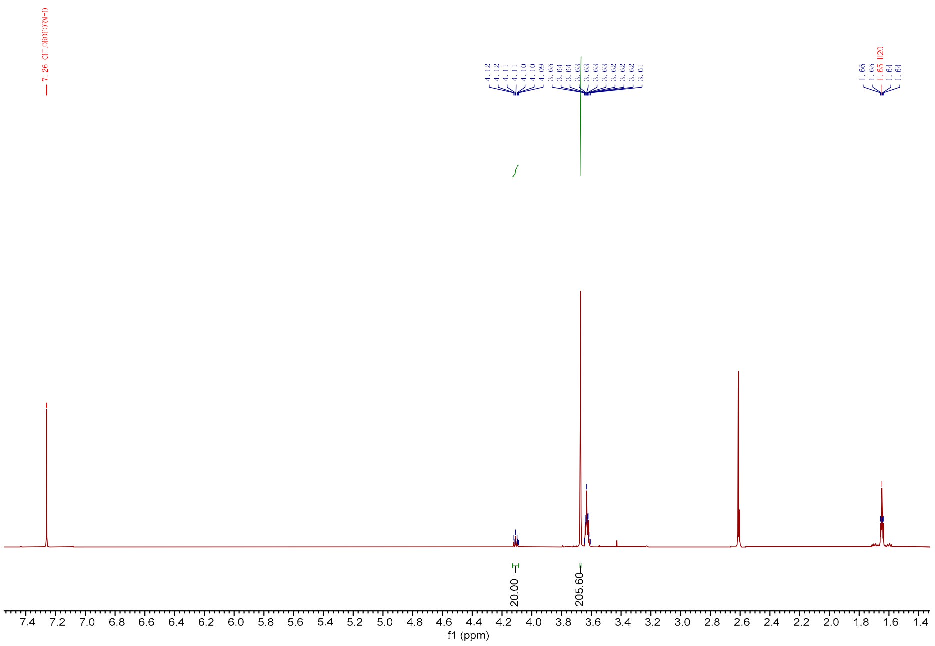

Figure S16. ^{1}H NMR (400 MHz, $CDCl_3$) spectrum of TBD-catalyzed PBS depolymerization with 30 equiv. of methanol.

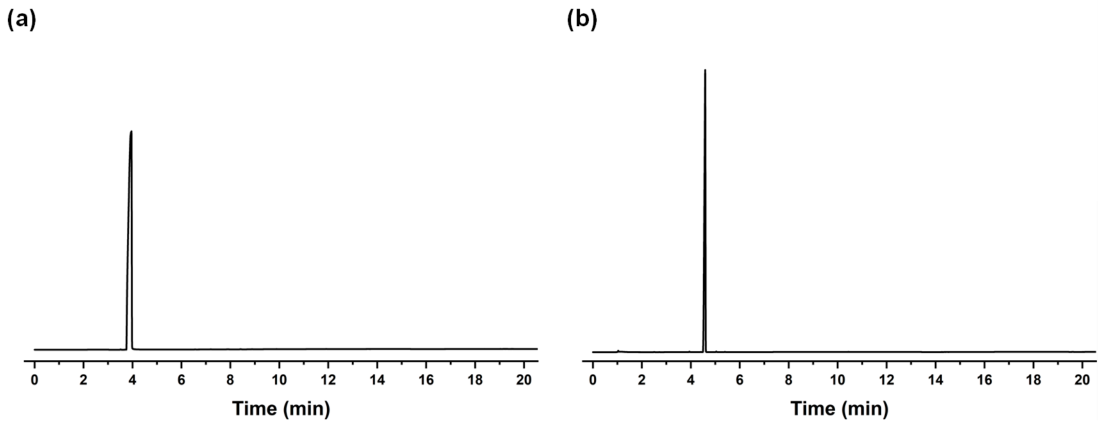

Figure S17 (a) GC chromatogram of 1,4-butanediol standard. (b) GC chromatogram of dimethyl succinate standard.

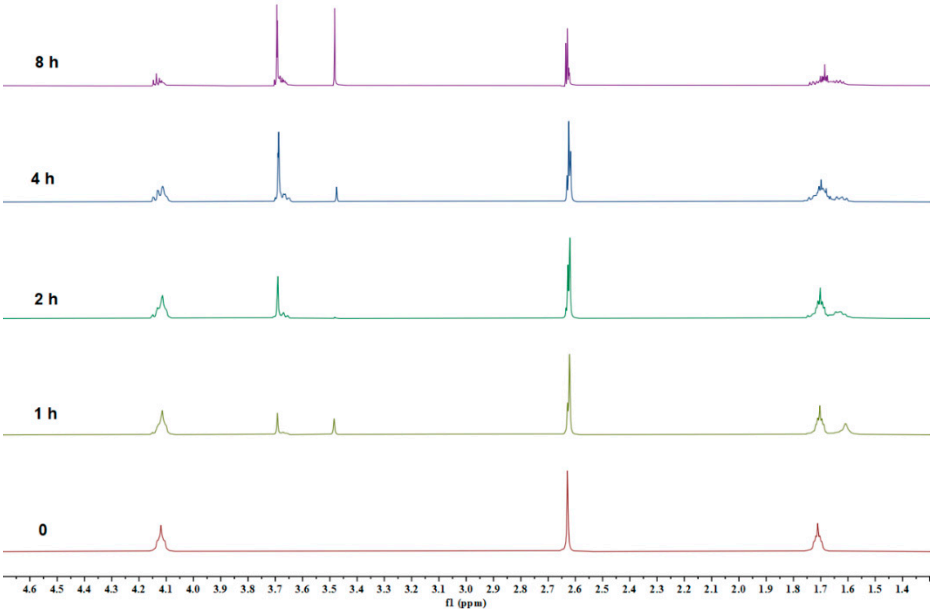

Figure S18. Stacked ^{1}H NMR spectra (400 MHz, $CDCl_3$) of PBS methanolysis catalyzed by TBD at 80 °C.

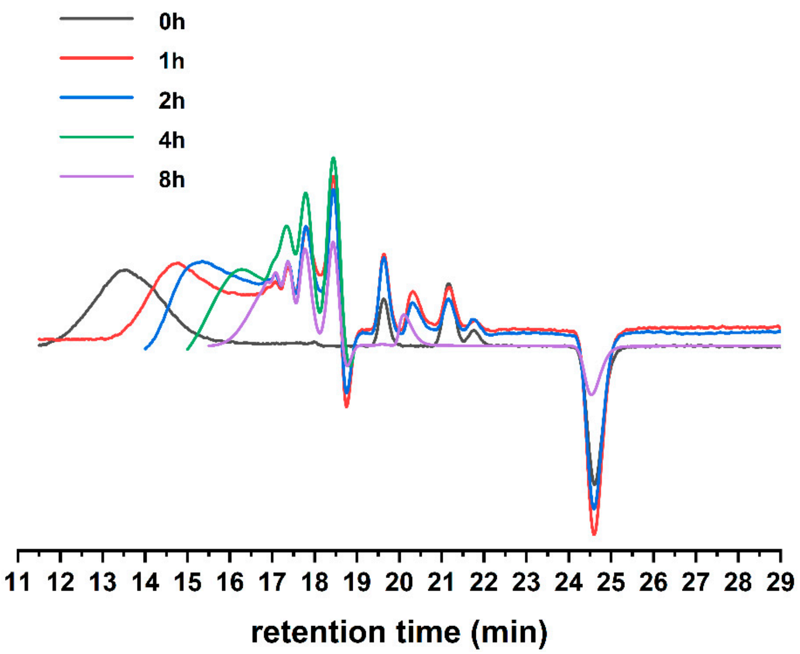

Figure S19. GPC curves recorded at different time points for TBD-catalyzed degradation of PBS at 80 °C.

Table S1. Depolymerization behavior study of PBS depolymerization using TBD[a]

Timepoint	M_n (g/mol)	M_w (g/mol)	M_v (g/mol)	PDI	Yield (%)[b]
0	57157	102393	83671	1.79	-
1h	17011	24877	22483	1.46	20.7
2h	8703	12117	13149	1.39	31.2
4h	4372	5182	4308	1.18	53.4
8h	1847	1934	1446	1.05	69.2

[a] General depolymerization conditions for kinetic studies: PBS (860mg, 10 mmol relative to the ester linkages, n_{MeOH}:n_{ester}:$n_{catalyst}$ = 10:1:0.01, Con.$_{(MeOH)}$=25M, at 80°C. [b] Determined by ^{1}H NMR spectroscopy.

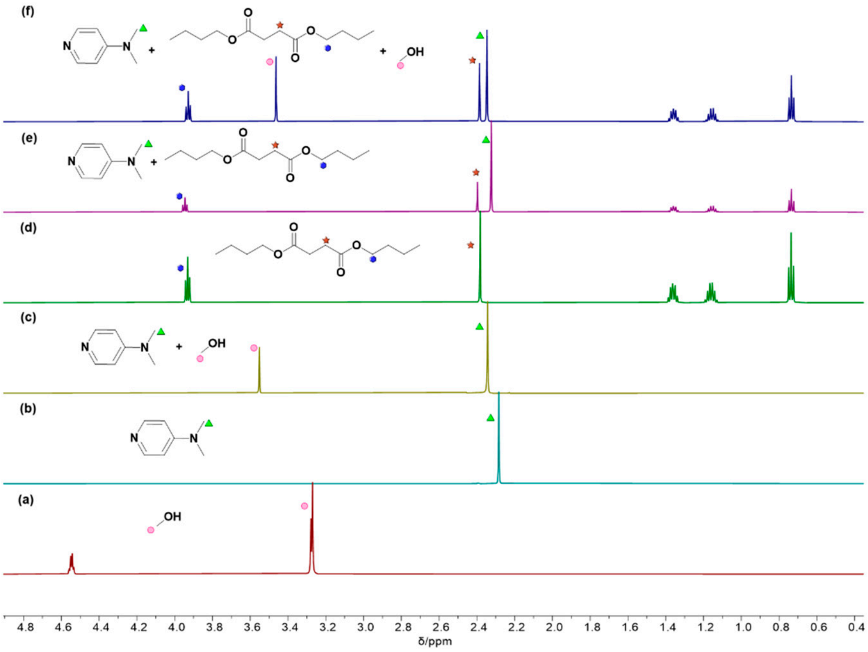

Figure S20. Stacked ^1H NMR spectra (C_6D_6, 500 MHz, 25 °C) of (a) methanol, (b) DMAP, (c) dibutyl succinate, (d) the mixture with equivalent amounts of DMAP and dibutyl succinate, (e) the mixture with equivalent amounts of DMAP, dibutyl succinate and methanol.
